# Stage‐Specific Responses to Warming in Trojan Fir Across Early Life Stages: Germination, Seedling Survival, and Seedling Growth

**DOI:** 10.1002/ece3.72774

**Published:** 2026-02-05

**Authors:** Nurbahar Usta, Çağatay Tavşanoğlu

**Affiliations:** ^1^ Institute of Science Hacettepe University Ankara Türkiye; ^2^ Department of Biology Hacettepe University Ankara Türkiye

**Keywords:** *Abies*, climate change, germination, Mediterranean montane forests, seedling survival and growth, Trojan fir

## Abstract

Understanding the early life‐stage responses of tree species to climate change is critical for predicting forest regeneration success and guiding conservation and management efforts. We investigated the effects of temperature, cold stratification, and light on germination and early seedling performance of *Abies nordmanniana* subsp. *equi‐trojani* (Trojan fir), an endangered endemic tree from north‐western Anatolia (Türkiye). Germination was tested under fixed (10°C, 15°C, 20°C, 25°C, 30°C) and alternating (15°C/25°C, 20°C/30°C) incubation temperatures with and without cold stratification. Early seedlings were monitored for 10 days under controlled, nutrient‐free agar conditions. Our results show that while higher fixed and alternating temperatures enhance germination, early seedling survival declined at the warmest temperature (30°C), and root growth peaked at 20°C and decreased at higher temperatures. Cold stratification significantly improved germination across all temperature regimes, reducing the need for warmer incubation temperatures to achieve high germination. Light had a limited effect on overall germination. These findings indicate stage‐specific responses to warming, as warmer conditions favor germination, whereas cooler conditions favor early seedling survival and root allocation. Consequently, successful regeneration assessments and conservation planning should consider both germination and early seedling stages, alongside local thermal contexts, when evaluating the impacts of climate change on Trojan fir.

## Graphical abstract

We investigated the effects of temperature, cold stratification, and light on germination and early seedling growth in *Abies nordmanniana* subsp. *equi‐trojani* (Trojan fir), an endangered endemic tree from northwestern Anatolia (Türkiye). Our results show that while higher fixed and alternating temperatures enhance germination, they also lead to increased seedling mortality and reduced root growth. Mediterranean high‐altitude tree species may exhibit complex responses to warming at their early life stages, highlighting the necessity of considering both germination and seedling stages in conservation planning under climate change.

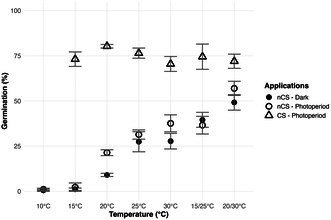

## ÖZET

Ağaç türlerinin iklim değişikliğine erken yaşam evrelerinde verdiği tepkileri anlamak, orman rejenerasyonunun başarısını öngörmek ve koruma ile yönetim çabalarını yönlendirmek açısından kritik öneme sahiptir. Bu çalışmada, kuzeybatı Anadolu'ya (Türkiye) endemik ve tehlike altındaki bir ağaç olan *Abies nordmanniana* subsp. *equi‐trojani* (Kazdağı göknarı) türünde sıcaklık, soğuk katlama ve ışığın çimlenme ve erken fide performansı üzerindeki etkileri incelenmiştir. Çimlenme, soğuk katlama uygulanmış ve uygulanmamış tohumlarda sabit (10°C, 15°C, 20°C, 25°C, 30°C) ve değişken (15°C/25°C, 20°C/30°C) inkübasyon sıcaklıklarında test edilmiştir. Erken fideler, kontrollü ve besinsiz agar koşullarında 10 gün süresince izlenmiştir. Bulgularımız, daha yüksek sabit ve değişken sıcaklıkların çimlenmeyi artırdığını; ancak en yüksek sıcaklıkta (30°C) erken fide sağkalımının azaldığını ve kök büyümesinin 20°C'de en yüksek değere ulaşıp daha yüksek sıcaklıklarda düştüğünü göstermiştir. Soğuk atlama, tüm sıcaklık rejimlerinde çimlenmeyi anlamlı biçimde artırmış ve yüksek inkübasyon sıcaklıklarına duyulan gereksinimi azaltmıştır. Işığın nihai çimlenme yüzdeleri üzerinde etkisi sınırlı kalmıştır. Bu bulgular, ısınmaya verilen evreye özgü tepkileri işaret etmektedir; daha sıcak koşullar çimlenmeyi desteklerken, daha serin koşullar erken fide sağkalımı ve köke yönlendirmeyi desteklemektedir. Sonuç olarak, Kazdağı göknarında iklim değişikliği etkilerini değerlendirirken hem çimlenme hem de erken fide evrelerinin, yerel termal bağlamlarla birlikte, koruma ve yönetim planlamasında dikkate alınması gerekmektedir.

## 1 Introduction

Climate change presents a multi‐layered challenge to forest ecosystems in the coming decades. As global temperatures rise, many forest species are expected to shift their ranges either toward higher latitudes or altitudes to cope with changing climatic conditions (Parmesan 2006; Chen et al. 2011). Although forest stands exhibit some degree of plasticity, their growth and vitality are likely to decline due to shifts in the timing and duration of temperature fluctuations, coupled with alterations in water availability (Resco de Dios et al. 2007). The longer generation times of trees compared to other plant species pose an additional limitation on the ability of forests to adapt rapidly to environmental change (Savolainen et al. 2007; Alberto et al. 2013). Mediterranean forests are among the most vulnerable ecosystems to climate change, facing significant risk to their stability and resilience (Hoffmann et al. 2008; Valladares et al. 2014; Nunes et al. 2021). At the same time, many Mediterranean woody plants may be relatively less affected by rising temperatures as they are well adapted to warm and dry conditions (Ogaya and Peñuelas 2021). However, tree species adapted to cooler temperatures, such as conifers inhabiting the mountainous belts of the Mediterranean, are particularly at risk, as these climatic shifts jeopardize their ecological stability and threaten their geographic distribution (Ruiz‐Labourdette et al. 2013). Their increased sensitivity to rising temperatures places cool‐adapted tree species in a precarious position under ongoing climate change (Lloret et al. 2004; Aldea et al. 2023; Hauck et al. 2025).

The impact of climate change on Mediterranean tree species has been widely investigated using species distribution models (SDMs) based solely on species presence and climate variables (Benito Garzón et al. 2008; Esteve‐Selma et al. 2010; Vessella et al. 2017; Almeida et al. 2022; Kassout et al. 2022). As in other coniferous trees, SDMs have projected shifts in the geographic ranges of circum‐Mediterranean firs (*Abies* spp.) under future climate scenarios (Sánchez‐Salguero et al. 2017; Usta Baykal 2019; Beridze et al. 2021; Méndez‐Cea et al. 2023; Vatandaşlar et al. 2023; López‐Tirado et al. 2024). However, these SDMs have rarely incorporated life‐history traits or fitness components (Benito Garzón et al. 2019). Because ambient temperature profoundly affects these species during early stages of their life cycle (Ramírez‐Valiente et al. 2021; Vicente and Benito‐Garzón 2024), models should integrate traits such as seed dispersal, germination, and seedling performance to better understand seedling establishment patterns under climate change. Therefore, experimental data on life‐history traits, including germination and early seedling growth, are urgently needed to improve predictions of Mediterranean fir responses to climate change (Aussenac 2002; Usta and Tavşanoğlu 2023).

Many conifers, including *Abies*, exhibit physiological seed dormancy that is broken by cold stratification, making them dependent on cold periods for successful germination and therefore sensitive to changes in winter conditions (Bewley and Black 2013; Baskin and Baskin 2014). For instance, *Abies pinsapo* exhibits declining germination with increasing temperature, suggesting that warming may negatively affect these species (Bravo‐Navas and Sánchez‐Romero 2022). Similarly, cold stratification enhances germination in *Abies nordmanniana*, whereas higher temperatures have been shown to reduce germination success (Tilki 2004). Moreover, germination responses to light or darkness can provide valuable information on seedling establishment strategies in canopy gaps versus understory environments (Xia et al. 2016; Hoyle et al. 2023). Light requirements during germination are also closely tied to early seedling growth and competitive strategies among tree species (González‐Rivas et al. 2009; Bravo‐Navas and Sánchez‐Romero 2022). However, the effect of light availability on germination within *Abies* remains poorly studied at the global scale, and the limited available evidence indicates highly species‐specific responses (Baskin and Baskin 2014). This substantial knowledge gap constrains our understanding of regeneration strategies in *Abies* species and their functional roles in forest succession across different biomes. More broadly, the lack of empirical data on the early life‐stage responses of circum‐Mediterranean firs to warming limits our capacity to anticipate their regeneration dynamics and resilience under future climatic conditions.

Trojan fir, *Abies nordmanniana* (Stev.) subsp. *equi‐trojani* (Aschers. & Sint. ex Boiss) Coode et Cullen, is an endemic coniferous tree taxon among many circum‐Mediterranean firs, with a natural distribution restricted in mid‐ and high‐elevations at mountain belts in northwestern Anatolia (Türkiye). Despite its endemic status and classification as Endangered by the IUCN (Knees and Gardner 2011), forestry practices have intensified, with harvest rates tripling over the past two decades (GDF 2023). The ecological characteristics and functional traits of the Trojan fir remain highly understudied, and its conservation is further hindered by decades of taxonomic uncertainty, complicating efforts to protect this vulnerable species (Usta and Tavşanoğlu 2023). The germination and seedling growth patterns of Trojan fir are highly unknown. Based on a few studies to date, Trojan fir displays among‐population variation in seed germination percentages due to catalase activity, explained by genetic variation and time of seed collection (Ak et al. 2012; Kurt et al. 2016). In another study, incubation at different temperatures (8°C, 12°C, 16°C, and 20°C) after 20 days of cold stratification did not change germination percentage, but higher temperatures resulted in faster germination (Yılmaz et al. 2011). Yılmaz et al. (2011) additionally noted this species can germinate even under the snow cover. Trojan fir has a high shade tolerance and does not require open gaps to grow, but especially in uneven‐aged stands, it may require some light for better seedling performance (Kara and Topaçoğlu 2018). Moreover, the sapling growth performance of Trojan fir is higher at high altitudes compared to low altitudes (Özden Keleş 2020), showing that the species performs better at cooler climatic conditions.

In this study, we investigated the effects of temperature, cold stratification, and light on the germination of Trojan fir, followed by assessments of early seedling mortality under different temperatures to estimate overall early life‐stage survival. We also measured early seedling growth based on root, shoot, and leaf lengths, and calculated derived traits such as the root/shoot and belowground/aboveground ratios. Because Trojan fir is native to cool, high‐altitude Mediterranean mountain belts and typically grows well under forest canopies, we hypothesized that lower temperatures, cold stratification, and dark conditions would promote germination, whereas higher temperatures and photoperiod conditions would suppress it. Based on the species' known climatic requirements, we further expected rising temperatures to increase seedling mortality and growth performance. Through these germination and early seedling growth experiments, we aimed to address key knowledge gaps in understanding the early‐life stage ecological requirements of this endangered species, providing essential insights for future modeling efforts and adaptive conservation strategies under climate change.

## 2 Methods

### 2.1 Seed Material

We obtained seed material from Eskişehir Forest Nursery Directory (Türkiye), originally collected from the natural population in Gürgendağ, western Anatolia (39.75′ N, 26.93′ E; 1300 m) by Balıkesir Forest Management Unit in 2020. Seeds were dried and then kept at −18°C for 2 years by the Eskişehir Forest Nursery Directory and 4°C in our laboratory for an additional 6 months until the experiments initiated. Seed mass was measured using a digital balance by weighing randomly selected 50 individual seeds (without wings), and the mean (±SE) was 66.0 ± 2.0 mg.

The seed collection site has a cool, weakly dry‐summer Mediterranean (Csb‐type) climate, representing a local high‐altitude variant within a region dominated by a warmer Mediterranean regime (Evrendilek et al. 2007; Türkeş and Yurtseven 2025). It has a mean annual temperature of 8.6°C and an annual total precipitation of 804 mm, with modest summer mean daily maxima (July–August *T*_max_ = 22°C) and below‐freezing winter mean daily minima (January–February *T*_min_ = −2.4°C) (Table S1; data from WorldClim v2.1; Fick and Hijmans 2017).

### 2.2 Germination Experiments

#### 2.2.1 Temperature Experiment

We started the germination experiments under five constant and two alternate temperatures (10°C, 15°C, 20°C, 25°C, 30°C and 15°C/25°C, 20°C/30°C), two different light treatments. Since the effect of light on germination is not known for Trojan fir and was studied for only a limited number of *Abies* species (Baskin and Baskin 2014), we applied both 24 h constant dark and 12 h:12 h photoperiod treatments in each incubation temperature. The germination experiments were conducted in temperature‐ and light‐controlled climate cabinets (Nüve TK252). For each treatment, 50 seeds were sown in four Petri dishes with pure (nutrient‐free) agar (0.7%) serving as replicates; thus, with seven temperature and two light treatments, a total of 72 Petri dishes with 3600 seeds were used in the temperature experiment. We performed our experiments in pure agar since it provides a chemically inert, moisture‐retaining medium to avoid providing extraneous nutrients and thereby isolate the effects of the treatments on germination, making it ideal for germination and early seedling growth experiments.

#### 2.2.2 Cold Stratification Experiment

At the same time as the temperature experiment, we also employed a cold stratification experiment following the same protocol for seed number and replication. Petri dishes containing 50 seeds each on pure nutrient‐free agar (0.7%) were kept at 4°C for 35 days. After the cold period, the dishes were transferred to six different incubation temperature regimes (15°C, 20°C, 25°C, 30°C, 15°C/25°C, and 20°C/30°C) under a 12 h:12 h photoperiod. Since the cold stratification and temperature experiments were initiated simultaneously, some early germination results from the latter were already available when the cold‐stratified seeds were transferred to incubation temperatures. These preliminary results, showing no germination at 10°C and similar responses under dark and photoperiod conditions, led us to exclude 10°C and dark treatments from the stratification experiment to reduce workload. Seeds that did not undergo stratification but were otherwise exposed to the same temperature and light conditions served as controls for each corresponding temperature treatment in the cold stratification experiment.

#### 2.2.3 Germination Checks

We checked germinations at three‐day intervals and seeds were considered germinated when the radicle had emerged to a length of at least 0.5 mm. For the group subjected to complete dark conditions, the counting process was carried out under the green light in a dark room to prevent photosynthetically active light exposure. For each counting session, the germinated seeds were removed from the Petri dishes, the number of germinated seeds was recorded, along with the corresponding date, to maintain accurate and detailed records of the germination timeline for analysis. The germination experiment was conducted over a 60‐day period and concluded once no new germination was observed for 15 consecutive days in any Petri dish.

#### 2.2.4 Seed Viability Assessment

To accurately quantify germination percentages, we performed a cut test on all non‐germinated seeds to distinguish non‐viable seeds (i.e., empty seeds without an embryo or rotten ones) from viable but ungerminated (i.e., dormant) ones in each dish. Accordingly, empty seeds were subtracted from the initial 50 seeds per Petri dish when calculating germination percentages.

### 2.3 Early Seedling Experiment

We performed an early seedling experiment to monitor and record the mortality and growth of seedlings under different incubation temperature conditions for the next 10 days after their germination. To ensure that mortality reflected temperature effects rather than nutrient limitation on the nutrient‐free agar, we restricted observations to the first 10 days; in preliminary checks, mortality beyond Day 10 increased across treatments irrespective of temperature, indicating confounding by nutrient depletion (see Figure S1, for representative seedlings). We sowed 350 seeds in seven Petri dishes (50 seeds per dish) containing pure nutrient‐free agar (0.7%) and placed them at 20°C/30°C alternate temperature under photoperiod (12 h:12 h) to germinate, which are optimal conditions for germination in the study species as suggested by the results of our germination experiment. We performed early seedling experiment under four incubation temperatures (20°C, 25°C, 30°C, and 20°C/30°C) with 12 h:12 h photoperiod conditions for 10 days. We did not use other incubation temperatures that we included in our germination experiment since germination remained minimum at those conditions.

#### 2.3.1 Seedling Transfer Following Germination

We checked germination daily, and seeds were immediately transferred to the designated incubation temperatures on the day germination occurred. Each germinated seed was carefully transferred to an individual Petri dish containing pure nutrient‐free agar (0.7%) to monitor subsequent growth. The main criterion for seedling transfer was that seeds had just germinated, with the radicle not exceeding 0.5 mm in length. To avoid confounding effects arising from emergence timing, germinated seeds were evenly and randomly allocated across all temperature treatments on each check day. Overall, there was a week difference between the first and last seedlings transferred. In this way, we successfully distributed seedlings among temperature treatments, with initial numbers ranging from 45 to 46 per treatment. Because we used pure agar as the growth medium, we eliminated potential confounding effects of nutrient addition on seedling survival and growth. We also limited the early seedling experiment to 10 days to ensure that any observed mortality could be attributed to temperature effects rather than nutrient depletion. The end of the 10‐day period (i.e., the measurement day) was defined individually for each seedling, as germination occurred within a one‐week range.

#### 2.3.2 Early Seedling Mortality

Early seedling mortality was estimated by direct counts of viable and non‐viable (dried or rotten) seedlings on the 5th and 10th days of the experiment, with non‐viable individuals removed during both assessments.

#### 2.3.3 Early Life‐Stage Survival

Using early seedling mortality percentages on the 10th day and the corresponding germination percentages at each temperature, we estimated early life‐stage survival ratio for each temperature treatment. Overall early‐life stage survival (*S*) was calculated using the formula:

$$S=G\times \left(1-M÷100\right),$$

where *G* represents germination (%) at the respective temperature, and *M* represents early seedling mortality (%).

#### 2.3.4 Early Seedling Growth

On the 10th day following germination, we measured three traits for each seedling: root length, shoot length, and leaf length. Shoots and roots displayed distinct differences in color and tissue texture, with leaves emerging as dark green from the top of the shoot (Figure S1). Each seedling was dissected into root, shoot, and leaf components, and measurements were taken using a ruler with millimeter precision. A small number of seedlings had curled roots, which were gently straightened before measurement. While root length, shoot length, and leaf length are direct measurements in cm, root/shoot ratio (RS) and belowground/aboveground ratio (BA) were calculated using the following formulas:

RS=Root Length÷Shoot Length

$$\mathrm{BA}=\text{Root Length}÷\left(\text{Shoot Length}+\text{Leaf Length}\right)$$

### 2.4 Data Analysis

Prior to germination analysis, we corrected the germination data by removing empty seeds (as determined by the cut test at the end of the experiment) from the dataset of germinable seeds. We then used generalized linear models (GLMs) with a binomial error distribution to analyze germination outcomes, as this approach is appropriate for binary data (germinated vs. ungerminated) and has been widely applied in germination studies (e.g., Burghardt et al. 2016; Ghaderi‐Far et al. 2021, among others). For the temperature experiment, we fitted the model “Germination ~ Temperature × Light”, which included both main effects and their interaction. For the cold‐stratification experiment, we used the model “Germination ~ Temperature + Stratification”, including both factors as main effects. In each case, a likelihood ratio test compared the null model with those containing the specified fixed factors.

For seedling growth analyses, we assessed how incubation temperature affected early seedling traits, including root length, shoot length, and leaf length, as well as root/shoot and belowground/aboveground ratios. For each trait, we applied a GLM with a Gaussian error distribution using the model “Trait ~ Temperature”, treating temperature as a fixed factor.

Post hoc multiple comparisons among temperature treatments were conducted using estimated marginal means for both germination and seedling growth analyses. After each analysis, model residuals were visually inspected for assumption violations (e.g., overdispersion or lack of fit). We performed all analyses in R (R Core Team 2021) and used the “emmeans” package (Lenth 2020) for estimating marginal means following GLMs and the “ggplot2” package (Wickham 2016) for graphical outputs.

## 3 Results

### 3.1 Germination

**FIGURE 1:**
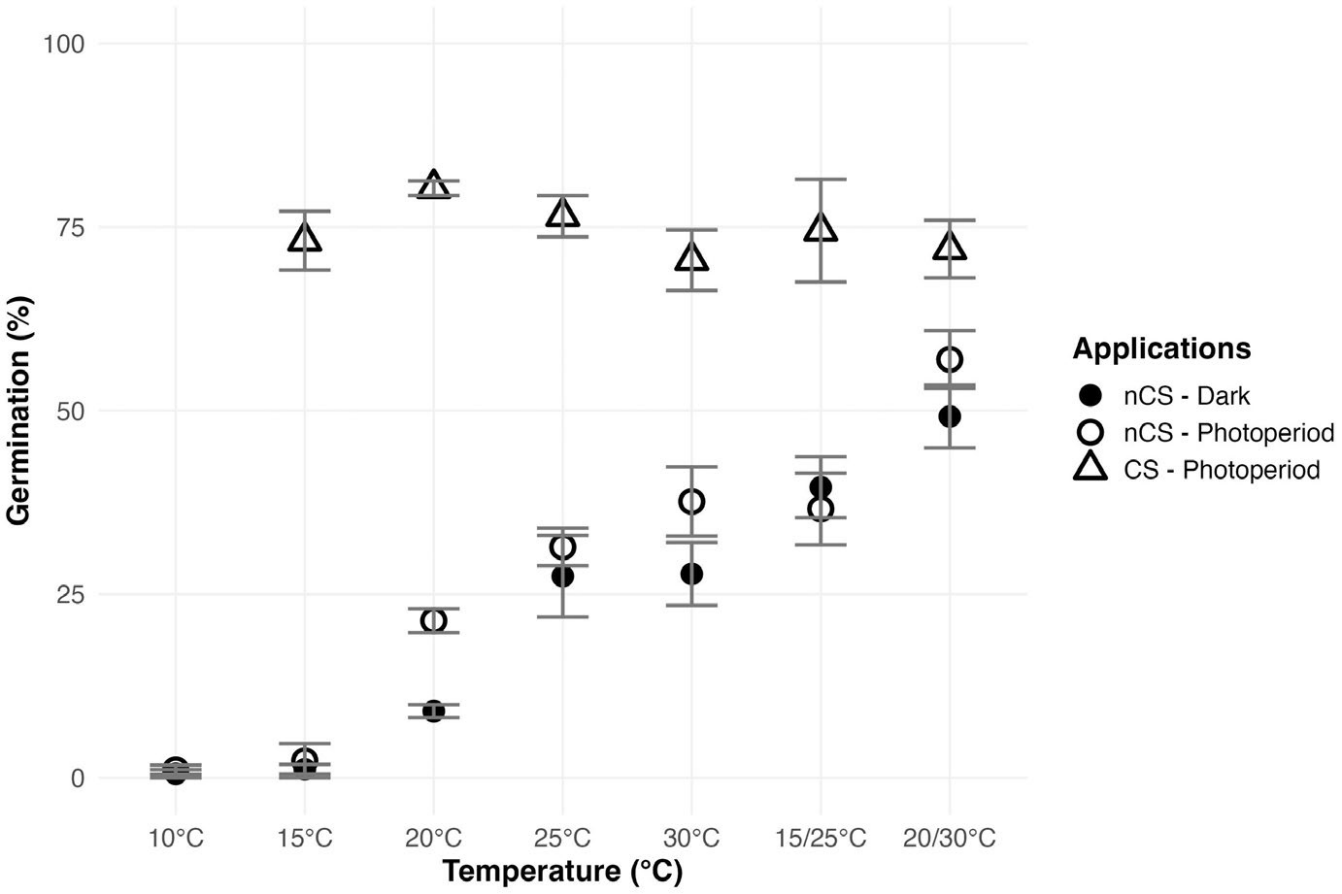
Mean (±SE) germination percentages of Trojan fir seeds under different constant (10°C, 15°C, 20°C, 25°C, and 30°C) and alternating (15°C/25°C and 20°C/30°C) temperatures, different light conditions (12 h:12 h photoperiod and complete dark), and cold stratification.

Germination percentages varied across temperature treatments (explained deviance [ED] = 87.4%, *p* < 0.0001; Table 1; Figure 1). We observed very limited germination at 10°C and 15°C under photoperiod and dark conditions. At higher temperatures, seeds exposed to photoperiod conditions exhibited improved germination, yet the effect of light was insignificant overall (ED = 1.0%, *p* > 0.05; Table 1). The highest percentages observed at 20°C/30°C reached over 50% (ED = 90.4%, *p* < 0.0001; Table 2). Alternating temperatures also resulted in different significances in germination percentages compared to their fixed counterparts. Specifically, germination percentages at 20°C/30°C were significantly higher than 25°C, respectively, in both darkness and photoperiod conditions (*p* < 0.0001; Table 2), yet the difference between 20°C and 15°C/25°C in photoperiod condition was insignificant (*p* = 0.09; Table 2).

**TABLE 1:** Summary of statistical analysis (likelihood ratio test; GLM) of the effect of incubation temperature and light on the germination of Trojan fir.

Factor	df	*χ* ^2^	Explained deviance (%)	*p*
Temperature	6	515.5	87.4	< 0.0001
Light	1	6.0	1.0	0.015
Temperature × Light	13	533.3	90.4	< 0.0001

**TABLE 2:** The results of pairwise comparisons (*p* values) of germinations between different incubation temperatures under different light regimes in Trojan fir.

Light	Temperature	15°C	20°C	25°C	30°C	15°C/25°C	20°C/30°C
Photoperiod	10°C	0.99	< 0.01	< 0.0001	< 0.0001	< 0.0001	< 0.0001
	15°C	—	< 0.0001	< 0.0001	< 0.0001	< 0.0001	< 0.0001
	20°C		—	0.65	0.04	0.09	< 0.0001
	25°C			—	0.90	1.00	< 0.0001
	30°C				—	1.00	0.03
	15°C/25°C					—	0.01
Dark	10°C	0.98	0.28	< 0.01	< 0.01	< 0.001	< 0.0001
	15°C	—	0.23	< 0.001	< 0.001	< 0.0001	< 0.0001
	20°C		—	< 0.01	< 0.01	< 0.0001	< 0.0001
	25°C			—	1.00	0.56	< 0.01
	30°C				—	0.57	< 0.01
	15°C/25°C					—	0.89

Seeds treated with cold (4°C for 35 days) under photoperiod conditions consistently exhibited the highest germination percentages, reaching maximum levels (between 70.7% and 83.3%) among our full range of experiments, with no significant differences across incubation temperatures (ED = 3.7%, *p* > 0.05, Table 3). Cold stratification significantly improved germination at all incubation temperatures that the effect of cold stratification was tested (ED = 91.0%, *p* < 0.0001). Light treatments alone (dark or photoperiod) had a limited effect on overall germination percentages (ED = 1.0%, *p* = 0.015; Table 1; Figure 1) compared to the incubation temperature, yet the interaction between light and temperature showed a significant effect on germination percentage (ED = 90.4%, *p* < 0.0001; Table 1).

**TABLE 3:** Summary of the statistical analyses (likelihood ratio test; GLM) of the effect of cold stratification on germination in Trojan fir at different incubation temperatures.

Temperature	Estimate	SE	*p*
15°C	4.764	0.536	< 0.0001
20°C	2.709	0.285	< 0.0001
25°C	1.952	0.238	< 0.0001
30°C	1.349	0.232	< 0.001
15°C/25°C	1.585	0.242	< 0.001
20°C/30°C	0.671	0.254	0.025

*Note:* Each row indicates the comparison between cold stratification treatment and the corresponding control at a particular incubation temperature.

### 3.2 Early Seedling Mortality and Overall Early Life‐Stage Survival

**FIGURE 2:**
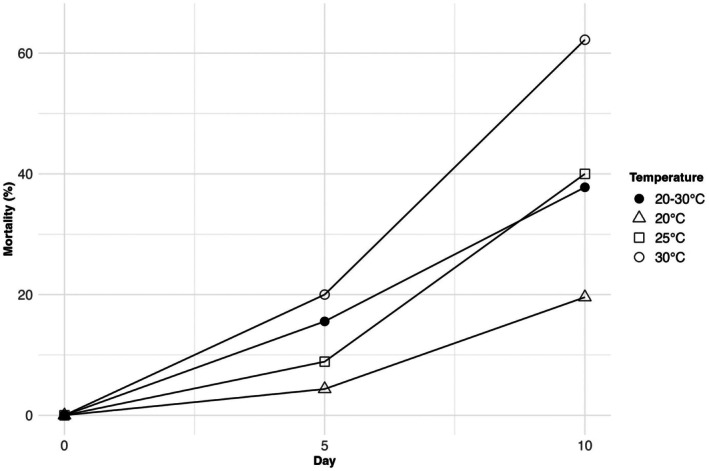
Mortality rates of seedlings on the 5th and 10th days after germination under four different temperatures (20°C, 25°C, 30°C, and 20°C/30°C) with 12 h:12 h photoperiod conditions.

Early seedling mortality percentage varied across temperature treatments over the 10‐day period (Figure 2). At 20°C, the mortality percentage remained the lowest throughout the experiment, increasing gradually but not exceeding 25% by Day 10. In contrast, higher temperatures showed greater increases in mortality over time. Particularly, at 25°C, mortality rose steadily, reaching approximately 40% by Day 10. The 30°C treatment and the alternating 20°C/30°C condition exhibited the highest mortality rates, exceeding 50% by Day 10, with similar trends observed in both treatments (Figure 2).

Early life stage survival, calculated from germination percentages and mortality rates at various temperatures, exhibited a decreasing trend as temperatures increased, except at the optimum germination temperature of 20°C/30°C, where it peaked at 22.8%. Survival rates at other temperatures were 19.1% at 20°C, 18.9% at 25°C, and 14.2% at 30°C (Table 4).

**TABLE 4:** Summary of the study results on germination, seedling mortality, and estimated overall early life stage survival.

Temperature	Germination (G%)	Seedling mortality (M %)	Overall early life stage survival (S%)
20°C	21.4	10.9	19.1
25°C	31.4	40.0	18.9
30°C	37.6	62.2	14.2
20°C/30°C	57.0	60.0	22.8

*Note:* Germination (%), seedling mortality (%) at the 10th day, and overall survival (%) during the early life stage (calculated using germination and seedling mortality) under different incubation temperatures are given.

### 3.3 Early Seedling Growth

**FIGURE 3:**
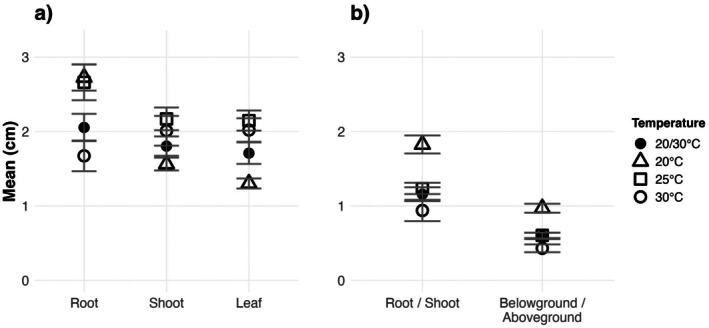
Different measurements of seedlings under four different temperatures (20°C, 25°C, 30°C, and 20°C/30°C) with 12 h:12 h. (a) shows the mean (±SE) root, shoot and leaf lengths; (b) shows the mean (±SE) root/shoot and belowground/aboveground ratios. Measurements were done on 35 seedlings at 20°C, 31 at 25°C, 19 at 30°C and 20 at 20°C/30°C due to different mortality rates in each group.

The growth responses of roots, shoots, and leaves, as well as root/shoot and belowground/aboveground ratios, also varied across temperature treatments (Figure 3; Table 5). Roots under 20°C treatments were longer than those under 30°C (Figure 3; Table 6; *p* < 0.001), whereas shoots at 20°C were shorter than at 25°C (*p* < 0.01), and leaves at 20°C were shorter than at 25°C and 30°C (Figure 3; Table 6; both *p* < 0.0001). Root/shoot ratios and belowground/aboveground ratios followed a similar trend; both ratios at 20°C were significantly greater than all other treatments (Figure 3; Table 6).

**TABLE 5:** Summary of statistical analysis (likelihood ratio test; GLM) of the effect of incubation temperature on the early root, shoot, and leaf growth of Trojan fir seedlings.

Growth parameter	*χ* ^2^	Explained deviance (%)	*p*
Root length	18.4	13.3	< 0.0001
Shoot length	8.7	17.3	< 0.0001
Leaf length	13.5	27.1	< 0.0001
Root/Shoot ratio	12.5	26.7	< 0.0001
Belowground/Aboveground ratio	4.5	37.7	< 0.0001

*Note:* Separate models were run for each parameter (df = 3 in all models).

**TABLE 6:** The results of pairwise comparison of early seedling growth in Trojan fir between different incubation temperatures.

Part	Temperature	25°C	30°C	20°C/30°C
Root	20°C	0.99	< 0.01	0.12
	25°C	—	0.01	0.21
	30°C		—	0.69
Shoot	20°C	< 0.001	0.11	0.63
	25°C	—	0.40	0.04
	30°C		—	0.75
Leaf	20°C	< 0.0001	< 0.001	0.02
	25°C	—	0.88	0.19
	30°C		—	0.66
Root/Shoot ratio	20°C	< 0.001	< 0.0001	< 0.001
	25°C	—	0.29	0.96
	30°C		—	0.64
Belowground/Aboveground ratio	20°C	< 0.0001	< 0.0001	< 0.001
	25°C	—	0.12	0.99
	30°C		—	0.21

## 4 Discussion

Our experiments show that temperature and cold stratification are the primary drivers of germination in Trojan fir, whereas light plays a minor role relative to temperature. Cold stratification markedly enhanced germination across all incubation temperatures. Without stratification, germination was limited at 10°C–15°C but increased at ≥ 20°C, with alternating regimes (20°C/30°C) further boosting germination. In contrast, early seedling mortality rose with increasing temperature, while growth (root, shoot, and leaf lengths and derived belowground: aboveground allocation ratios) generally increased under warmer temperatures. Consequently, our overall early life‐stage survival metric (integrating germination and day‐10 mortality) tended to be highest at cooler to intermediate temperatures, indicating a mismatch between thermal conditions that maximize germination and those that favor early survival. Taken together, these patterns indicate stage‐specific thermal strategies in early life (Barton 2024), with warmer regimes favoring germination and cooler regimes favoring early seedling survival and root allocation.

Enhanced germination after cold stratification at all incubation temperatures tested aligns with evidence that exposure to cold is a well‐established cue for stimulating germination in many temperate and high‐elevation species (Giménez‐Benavides et al. 2005). Across *Abies*, cold stratification is also required for germination (Baskin and Baskin 2014). For example, cold stratification improves germination in *A. nebrodensis* (Scialabba 2019), and a strong interaction between cold stratification and incubation temperature has been reported for *A. marocana* (Hatzilazarou et al. 2021). Varying cold stratification durations also enhanced germination at both lower and higher incubation temperatures in *A. fraseri* (Adkins et al. 1984) and *A. nordmanniana* (Tilki 2004). Our results confirm that cold stratification breaks dormancy and maximizes germination in Trojan fir (up to ~80%), even under suboptimal incubation temperatures (15°C–20°C). Notably, under stratification, germination remained relatively high across all incubation temperatures tested, which is noteworthy for *Abies* given the species‐specific thermal niches reported in the genus (Tilki 2004; Baskin and Baskin 2014; Hatzilazarou et al. 2021). By contrast, limited germination at 10°C and 15°C without stratification indicates suboptimal thermal conditions for dormancy release. Whereas germination increased at ≥ 20°C, and alternating regimes (20°C/30°C) further enhanced germination, consistent with temperature fluctuations acting as a cue for favorable establishment conditions. Collectively, these findings indicate that cold stratification and incubation temperature are critical determinants of germination in Trojan fir. These patterns are consistent with the native high‐altitude Mediterranean setting, where prolonged winter cold and transitional‐season temperature fluctuations are common (Lionello et al. 2006). The requirement for a preceding cold period to break physiological dormancy implies that reduced winter chilling under warming could constrain dormancy release and narrow the window in which adequate chilling and suitable post‐chilling temperatures co‐occur (Walck et al. 2011). Beyond Trojan fir, similar constraints may emerge from narrowly distributed, high‐elevation species whose germination depends on winter chilling (Baskin and Baskin 2014). Thus, while warmer post‐chilling conditions may promote germination in Trojan fir, diminished winter chilling can still curtail recruitment at warmer sites and displace successful regeneration to cooler microclimates or higher elevations, consistent with microclimatic buffering and upslope shifts reported for many species and ecosystems (Scherrer and Körner 2011; Maclean et al. 2015; De Frenne et al. 2019).

Higher early‐seedling mortality at warmer incubation temperatures contrasted with the germination responses, indicating stage‐specific thermal sensitivity. Placed in the context of the seed‐source climate (Table S1), the intermediate/alternating temperatures (20°C–25°C; 20°C/30°C) sit at or slightly above typical summer daily maxima, whereas 30°C lies beyond that envelope, matching the observed drop in early survival at 30°C. In line with this pattern, sapling performance in the field is higher at high (cooler) elevations than at low (warmer) elevations in Trojan fir (Özden Keleş 2020). Elevated temperatures increase respiratory costs and can push plants toward negative carbon balance (Atkin and Tjoelker 2003; Ow et al. 2008), while warming also exacerbates water stress by narrowing hydraulic safety margins and increasing evapotranspirative demand (Rennenberg et al. 2006; Choat et al. 2012). Such effects particularly threaten small seedlings with limited root systems (Grossnickle 2012). Consistent with a stage‐specific interpretation (Barton 2024), the seedling stage appears less generalist than germination (especially after stratification), showing sharper survival declines at the warmest temperature. Accordingly, overall early life‐stage survival peaked at 20°C–25°C and 20°C/30°C, but declined at 30°C. This pattern is most parsimoniously explained by the higher thermal sensitivity of the seedling stage: warmer temperatures can raise germination, yet seedling survival drops at the warmest temperature, consistent with known vulnerabilities of small seedlings under heat and water stress (Grossnickle 2012; Choat et al. 2012).

Root growth was the most temperature‐sensitive seedling trait, with maximum development at 20°C, indicating that relatively cooler temperatures promote belowground allocation that supports water and nutrient uptake during establishment (Binder et al. 1990; Schall et al. 2012; Song et al. 2021). Beyond resource acquisition, greater root allocation is associated with higher drought survival in Mediterranean seedlings. For instance, increased root investment explains the higher survival of *Quercus suber* relative to *Q. ilex* during summer drought in sandy soils (Ramírez‐Valiente et al. 2019), and field warming advanced emergence but increased seedling mortality in oaks, highlighting the value of traits that enhance water uptake under warmer conditions (Lázaro‐González et al. 2023). Consistent with this pattern, in conifers, seedling survival tightly tracks water relations and root system development, with establishment depending on roots capable of accessing reliable water during drought (Grossnickle 2012; Mackay et al. 2020). In our study, shoot and leaf growth varied less across treatments (slightly higher at 25°C), while the root: shoot ratio peaked at 20°C, suggesting prioritization of root development under cooler conditions. Taken together, and in line with our finding of maximal root development at 20°C, temperatures that foster root growth are likely to indirectly enhance drought resistance during establishment. Considering the local thermal baseline (Table S1), 20°C–25°C and 20°C/30°C fall within the usual warm‐season range, but 30°C lies outside it, which accords with the lower early survival at the warmest treatment. Recent syntheses indicate that warming effects on early life stages vary among biomes, species, and populations, with potential benefits where experimental optima meet or exceed local means but increasing risk near upper thermal limits (Sentinella et al. 2020; Vicente and Benito‐Garzón 2024), and warmer springs can advance germination phenology in Mediterranean trees. Accordingly, for Trojan fir, moderate warming near current summer conditions may maintain or modestly improve germination, yet increased seedling mortality at higher temperatures suggests a threshold beyond which net establishment could decline and become contingent on microclimate and moisture availability. More generally, whether warming aids or hinders early life stages depends on proximity to upper thermal limits (Sentinella et al. 2020; Vicente and Benito‐Garzón 2024).

Taken together with the local climate envelope (Table S1), the stage‐specific mismatch we observed (warmer temperatures favoring germination but depressing early survival at the warm end) warrants caution when interpreting projections of lower‐elevation expansion from correlative, climate‐only SDMs (Tekin et al. 2022; Ertürk et al. 2024). Other correlative studies forecast losses in distributional range (Usta Baykal 2019; López‐Tirado et al. 2024), underscoring the sensitivity of classic SDMs to algorithms and predictor sets (Elith and Leathwick 2009). We do not provide a formal model‐data comparison; rather, our experiments delineate an establishment‐relevant thermal window near current warm‐season conditions and indicate declining performance toward warmer extremes. Incorporating early life‐stage traits (germination, seedling survival) and local thermal baselines into trait‐ and demography‐informed SDMs should help reconcile these differences (Elith and Leathwick 2009; Ehrlén and Morris 2015).

## 5 Conclusions

Our experiments show that temperature and cold stratification are the dominant controls of Trojan fir regeneration, with light playing a minor role. Warmer and alternating regimes increased germination, whereas early seedling survival (and root allocation) declined toward the warm end; consequently, early life‐stage survival peaked at cooler‐intermediate temperatures, indicating stage‐specific thermal strategies. Interpreted in the context of the seed‐source climate, these responses suggest that establishment is favored near current warm‐season conditions but deteriorates at warmer extremes. These inferences are necessarily cautious because they derive from nutrient‐free agar and a short (10‐day) observation window under controlled conditions; soil moisture, substrates, and microsite heterogeneity may modify effect sizes in the field. From a conservation perspective, the key implication is that early life stages act as a thermal filter. Management and forecasting should therefore incorporate germination, seedling survival, and their local thermal baselines rather than relying solely on adult occurrences or climate correlations in SDMs. Practically, this points to evaluating cooler or shaded microsites, seasonally cooler planting windows, and the protection of high‐elevation refugia that enhance drought resistance during establishment; actions to be validated with field trials before broad application.

## Author Contributions

**Nurbahar Usta:** conceptualization (supporting), data curation (lead), formal analysis (lead), investigation (lead), software (lead), writing – original draft (lead), writing – review and editing (equal). **Çağatay Tavşanoğlu:** conceptualization (lead), methodology (lead), resources (lead), software (supporting), supervision (lead), writing – review and editing (equal).

## Funding

Nurbahar Usta was supported by the Higher Education Council of Türkiye (YÖK), 100/2000 Priority Areas Program.

## Conflicts of Interest

The authors declare no conflicts of interest.

## Supporting information


**Table S1:** Monthly mean (*T*_mean_), minimum (*T*_min_), and maximum (*T*_max_) temperatures and monthly total precipitation at the seed collection site. Annual means of temperature variables and annual total precipitation are also provided. Data are from WorldClim v.2.1 (Fick and Hijmans 2017).
**Figure S1:** Representative images of seedlings. (a) A seedling on the 10th day following germination; (b) dissected components; leaves, shoot, and root (left to right), separated prior to measurement.

**Data S1:** ece372774‐sup‐0002‐DataS1.zip.

## Data Availability

Raw data of germination and seedling growth experiments are available as Supporting Information.
