# Supplementary material for: Stage‐Specific Responses to Warming in Trojan Fir Across Early Life Stages: Germination, Seedling Survival, and Seedling Growth
Source: Ecol Evol. 2026 Feb 5;16(2):e72774. doi: 10.1002/ece3.72774 (PMC12875746; doi:10.1002/ece3.72774)
Supplement: Supplementary file 1 — Table S1: Monthly mean (T mean), minimum (T min), and maximum (T max) temperatures and monthly total precipitation at the seed collection site. Annual means of temperature variables and annual total precipitation are also provided. Data are from WorldClim v.2.1 (Fick and Hijmans 2017). Figure S1: Representative images of seedlings. (a) A seedling on the 10th day following germination; (b) dissected components; leaves, shoot, and root (left to right), separated prior to measurement. [file ECE3-16-e72774-s002.docx]

**SUPPORTING INFORMATION**

**Contrasting responses in germination and early seedling survival and growth to warming in Trojan fir**

**Table S1.** Monthly mean (T_mean_), minimum (T_min_), and maximum (T_max_) temperatures and monthly total precipitation at the seed collection site. Annual means of temperature variables and annual total precipitation are also provided. Data are from WorldClim v.2.1 (Fick and Hijmans 2017).

|  | **Temperature** | | |  |
| --- | --- | --- | --- | --- |
| **Month** | **T_mean_ (°C)** | **T_min_ (°C)** | **T_max_ (°C)** | **Precipitation (mm)** |
| January | 0.4 | -2.4 | 3.1 | 112 |
| February | 0.4 | -2.4 | 3.3 | 100 |
| March | 2.5 | -1.0 | 5.9 | 82 |
| April | 6.8 | 2.9 | 10.7 | 70 |
| May | 11.3 | 6.8 | 15.7 | 58 |
| June | 15.2 | 10.3 | 20.0 | 37 |
| July | 17.2 | 12.5 | 22.0 | 19 |
| August | 17.2 | 12.5 | 22.0 | 19 |
| September | 14.5 | 10.0 | 19.0 | 27 |
| October | 10.2 | 6.5 | 13.9 | 52 |
| November | 5.7 | 2.5 | 8.9 | 93 |
| December | 2.5 | -2.0 | 5.2 | 135 |
| **Mean** | **8.6** | **4.6** | **12.5** |  |
| **Total** |  |  |  | **804** |

**Reference**

Fick S.E., Hijmans R.J. (2017) WorldClim 2: new 1km spatial resolution climate surfaces for global land areas. *International Journal of Climatology* **37**(12), 4302-4315.


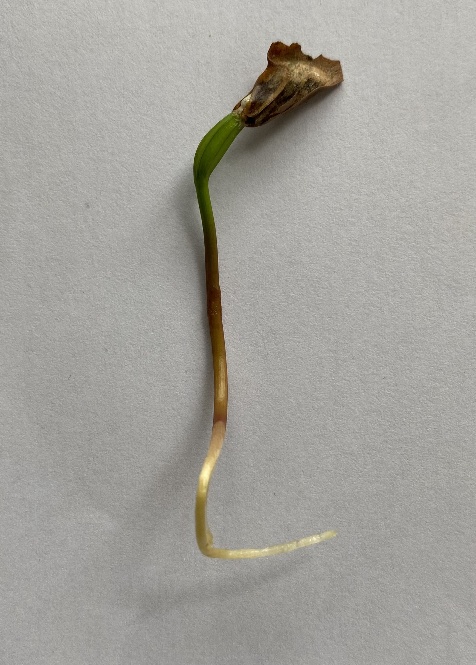

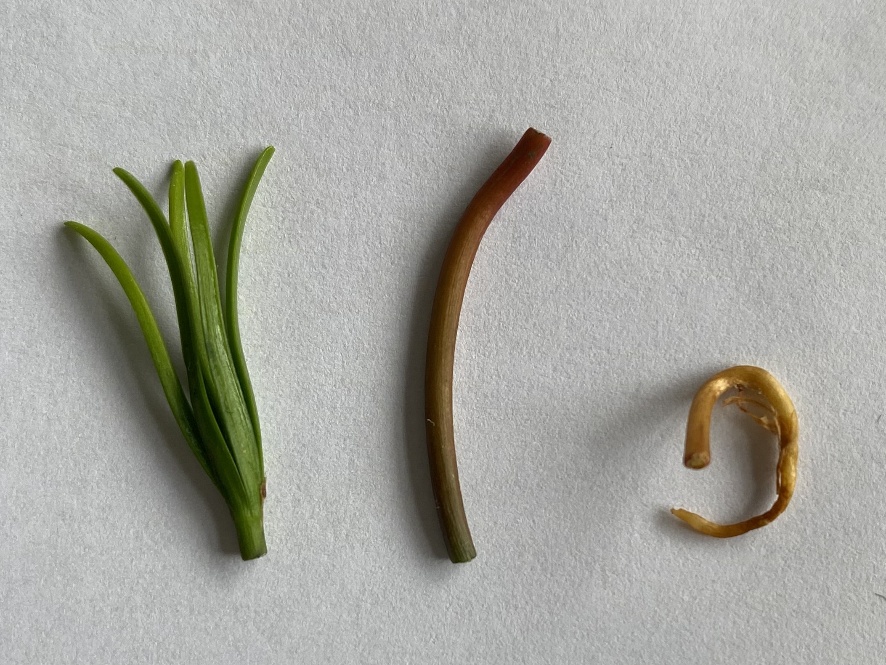


**Figure S1.** Representative images of seedlings. (a) A seedling on the 10th day following germination; (b) dissected components; leaves, shoot, and root (left to right), separated prior to measurement.
