# Supplementary material for: Stage‐Specific Responses to Warming in Trojan Fir Across Early Life Stages: Germination, Seedling Survival, and Seedling Growth
Source: Ecol Evol. 2026 Feb 5;16(2):e72774. doi: 10.1002/ece3.72774 (PMC12875746; doi:10.1002/ece3.72774)
Supplement: Supplementary file 2 — Data S1: ece372774‐sup‐0002‐DataS1.zip. [file ECE3-16-e72774-s001.zip › metadata.rtf]

METADATA FOR SUPPORTING INFORMATION

early_seedling_measurements: measurements of early seedlings on their 10th day
	id: seedling id
	temp: temperature the seedlings grown
	body: shoot length in cm
	root: root length in cm
	leaf: leaf length in cm
	num_leaf: number of needles (not included in the analysis)

early_seedling_mortalities: mortalities of early seedlings on 5th and 10th day
	temp: temperature the seedlings grown
	day: the day of the mortality measurement (5th or 10th)
	total: total number of seedlings

germination_non_viable: non-viable seeds after the cut test
	temperature: temperature
	light_treatment: dark or photoperiod
	replicate: replicate of a treatment (4 replicates each)
	non_viable: number of non-viable seeds (rotten or empty) in each replicate 

germination_amount: total number of germinated seeds in a replicate
	temperature: temperature
	light_treatment: dark or photoperiod
	replicate: replicate of a treatment (4 replicates each)
	germinated: total number of germinated seeds in each replicate

germination_raw: number of seeds germinated in each control day
	day: the control day 
	replicate: replicate of a treatment (4 replicates each)
	temp: temperature
	amount: the count of germinated seeds
